# Extracting Global Shipping Networks from Massive Historical Automatic Identification System Sensor Data: A Bottom-Up Approach

**DOI:** 10.3390/s19153363

**Published:** 2019-07-31

**Authors:** Zhihuan Wang, Christophe Claramunt, Yinhai Wang

**Affiliations:** 1 Institute of Logistics Science and Engineering, Shanghai Maritime University, Shanghai 201306, China; 2 Logistics Engineering College, Shanghai Maritime University, Shanghai 201306, China; 3 Naval Academy Research Institute, Brest 29240, France; 4 Civil & Environmental Engineering, University of Washington, Seattle, WA 98195, USA

**Keywords:** AIS big data, ship trajectory, shipping network, DBSCAN, stay locations, stop events

## Abstract

The increasing availability of big Automatic Identification Systems (AIS) sensor data offers great opportunities to track ship activities and mine spatial-temporal patterns of ship traffic worldwide. This research proposes a data integration approach to construct Global Shipping Networks (GSN) from massive historical ship AIS trajectories in a completely bottom-up way. First, a DBSCAN (Density-Based Spatial Clustering of Applications with Noise) algorithm is applied to temporally identify relevant stop locations, such as marine terminals and their associated events. Second, the semantic meanings of these locations are obtained by mapping them to real ports as identified by the World Port Index (WPI). Stop events are leveraged to develop travel sequences of any ship between stop locations at multiple scales. Last, a GSN is constructed by considering stop locations as nodes and journeys between nodes as links. This approach generates different levels of shipping networks from the terminal, port, and country levels. It is illustrated by a case study that extracts country, port, and terminal level Global Container Shipping Networks (GCSN) from AIS trajectories of more than 4000 container ships in 2015. The main features of these GCSNs and the limitations of this work are finally discussed.

## 1. Introduction

Shipping is an efficient and environmental-friendly transportation mode for long-distance transport of large quantity of goods [1]. It plays an important role in promoting international trade. According to the International Maritime Organization (IMO), more than 80 percent of world trade is transported by ships [2]. The concept of Global Shipping Network (GSN) models the global shipping traffic and cargo flows between ports. It is crucial for many maritime applications such as shipping route planning, network disaster or disruption evaluation, as well as invasive species spread at the global level.

A shipping network is usually developed from two different perspectives. One approach is explicitly based on service schedules as provided by big shipping companies such as Maersk, COSCO, and K-line, since these schedules are usually publicly accessible, and as the total number of ships accounts for a large percentage of the global maritime ships [3]. For instance, shipping schedule tables of 24 major carriers have been used to examine the spatial pattern and hub-spoke system of global shipping [3]. However, these schedules are notably incomplete and may not completely reflect ship delays, re-routing and void sailings [4]. Another way to construct a shipping network relies on the port arrival and departure records, which are usually provided by commercial data providers such as IHS global, MarineTraffic, FleetMon, and shipxy [5,6,7]. These records are usually extracted from AIS static messages but with much lower data quality than the dynamic messages of AIS data [8,9]. In addition, these port-level records cannot precisely derive terminal-level networks at a lower scale and then appropriate links with detailed operations of these ships. This research proposes a data integration bottom-up approach to construct shipping networks from massive dynamic AIS messages at a global scale. AIS messages contain rich ship movement information such as location, speed, and course [10], which together could reflect the global shipping network and cargo flows. Since ships usually stay at a given terminal for hours and send a large number of AIS messages, it would be possible to use some density-based unsupervised clustering method to identify terminal locations. In order to do so, we applied a DBSCASN (Density-Based Spatial Clustering of Applications with Noise) to identify terminal locations and ship stops. Terminal locations are further mapped to ports, cities, and countries as given by public datasets. Together with ship stops at each of these locations, we explore the travel sequences and shipping networks at multiple scale levels, and relate individual AIS locations to higher level locations such as terminals, ports, and countries.

The main motivation of this work is to build a primitive shipping network which can be applied to first analyze cargo flows at the global and regional scales. Second, this network allows for an analysis of ship behaviors and emissions at multiple levels, that is, from primitive AIS trajectories to terminal, port, and country levels. The multi-level network is made of a large set of ship trajectories which are semantically decomposed in sub-trajectories to reflect these multiples levels. The originality of our work is first to favor the generation of network node-to-node trajectories and then the potential derivation of main maritime routes and speed profiles for fuel consumption and ship emissions between two given terminals. The multi-network representation also supports additional network analysis. Centrality analysis are performed at the terminal, port, and country levels and characterize the main maritime nodes and routes, while the most important connections between maritime nodes are also identified.

The contributions of this research are as follows. First, it provides a novel approach to construct GSNs at multiple spatial levels, as well as a tree-based hierarchical structure to link bottom-level ship movements to high-level stop locations and shipping networks. Second, the approach develops a terminal level global shipping network based on AIS sensor data that provides a local view of the trajectory and stop patterns at the local port level. Third, this approach enables us to track the detailed movements and behaviors of each ship between any two terminals and ports, with this also being valuable for further applications evaluating ship emissions or extracting the main routes among terminals and ports.

The remainder of this paper is organized as follows. Section 2 reviews related works on trajectory mining, while Section 3 introduces the main datasets used in our study, and describes the main processes and methods used to develop shipping networks. Section 4 presents the case study and derivation of a terminal and port level global container-shipping network (GCSN). Finally, Section 5 sums up the main contribution and limitations of this research.

## 2. Related Work

Mobile object trajectories can be considered as a collection of time-ordered observations which contain rich spatial and temporal information [11]. With the increasing market penetration of GPS-enabled devices and availability of large-scale trajectory data, growing interests are found in extracting insights and knowledge from trajectories [12,13]. Detecting stops and stay locations from trajectories are fundamental tasks to facilitate a diversity of applications, such as mining travel sequences [14,15], travel recommendations [16,17], and destination prediction [18]. Spaccapietra et al. [19] introduce a conceptual data model which turns a raw trajectory into a stops-and-moves-of-trajectory (SMoT) by dividing trajectory points into a set of stop or move segments. Over the past years, several techniques have been developed to identify stops and moves. including IB-SMoT (Intersection Based SMoT) [20], CB- SMoT (Clustering based SMoT) [21], and DB-SMoT (direction based SMoT) [22].

The IB-SMoT method identifies stops by examining the intersection of each trajectory point with given potential stop regions. Trajectories within a region that exceed a minimal duration are labelled as stops. The CB-SMoT is a two-step method that first uses an unsupervised method to identify stop clusters and moves. Stop clusters that intersect a given region and satisfy minimal stay duration constraint are labelled as stops. The DB-SMoT approach takes the direction-variation of a trajectory into main consideration. It first calculates the direction variation of each pair of two sequential points in a trajectory and then cluster points based on the variation of direction and minimal time duration. Li and Zheng et al. [23] developed a similar algorithm, but it is based on distance and time duration among trajectory points.

DBSCAN has been widely used to identify stop points and stay locations detection. It performs relatively well to detect clusters with arbitrary shapes. It has proven to be computationally efficient for large datasets and also requires little inputs from domain knowledge [24,25]. Palma et al. [21] use DBSCAN to detect stops by clustering low speed points into a single trajectory. The original DBSCAN algorithm has been also extended to take both the temporal and spatial features into consideration [14,26]. Gong et al. [27] apply an improved version of DBSCAN (C-DBSCAN), which takes both time constraints and direction variations into account, and a Support Vector Machines (SVM) is applied to detect both stop status and stop types from trajectories. Density-based clustering methods are also used to mine parking locations [28] and travel sequences of mobile objects [15].

Although trajectory mining research is mainly applied to urban contexts [13], it appears with the increasing availability of ship trajectories, as extracting insights and knowledge from ship trajectories has drawn increasing attention. For example, Yan et al. [29] detect stay locations and main routes within the Port of Singapore from ship trajectories based on a modified DBSCAN which takes the location, speed, and direction of a vessel into account. Zhang et al. [30] investigate the spatial-temporal patterns of ship traffic in the port of Singapore based on large AIS datasets. De Souza et al. [31] develop an approach to identify fishing activity from Satellite-based AIS. Chen et al. [32] estimates the throughput of major container ports from containers ship trajectories and other open data sources. Ship trajectories are also used to identify ship stationary events and container handling events for measuring the performance of major container ports [33].

Although DBSCAN is indeed used in this study, the peculiarity of our approach is its multi-level component. In fact, most of current approaches are either applied at the port and local levels or at the global scale. For instance, several researches [29,30] have studied ship behaviors at the port level, but did not extend the principles of their approach to larger scales, as the motivation and application of these works is different from our work. Conversely, while several recent works investigate global shipping and maritime logistics and attempt to generate global shipping networks of different ship types based on port records or services schedules of major carriers, these studies do not consider local and global terminal-level transportation patterns or specific ship behaviors at the local level. We consider that many large ports, such as Shanghai, consist of several geographically separated terminals. This is an important property to analyze ship behaviors and activity at the local and regional levels.

Generally, and despite the specific interest and contribution of these works, the full potential of AIS data at both the global, regional and local scales is not completely addressed. In fact, current AIS data-driven researches are mainly oriented toward the analysis of trajectory patterns at either the regional and local levels. However, they generally cover a relatively small number of world ship fleet. In addition, they apply the analysis of world fleet patterns or global shipping trends, as well as local patterns at the port and terminal levels. Although a few recent works investigate global shipping and maritime logistics and attempt to generate some global shipping networks of different ship types based on port records or services schedules of major carriers, these studies still do not model local and global terminal-level transportation patterns, as well as specific ship behaviors at the local level. Instead, the objective of our research is to develop a multi-scale and hierarchical structure applied to large AIS data in order to generate global shipping networks and terminal-level networks. Overall, the advantage of our multi-level network is that it integrates the ship stop, terminal, port, and country levels, which is not only suitable for high-level statistics, analysis but also appropriate to perform low-level ship activity or behavior analysis.

## 3. Data and Methodology

### 3.1. Data

Developing a global shipping network requires a sound data integration approach from multiple datasets. The first dataset considered is given by a global AIS dataset that both includes coastal-based AIS and satellite AIS messages. This dataset contains the detailed and complete movement records of ships worldwide in 2015. Each record contains detailed information about its associated ship and journey. We mainly consider dynamic and geographical data from AIS dataset, including ship’s Maritime Mobile Service Identity (MMSI), a unique identification number, navigation status, Speed over Ground (SOG), Longitude, Latitude, and Timestamp-Coordinated Universal Time (UTC) second of the AIS message generated.

Ships usually send AIS messages every 2 to 10 seconds when they are at underway (the faster the speed and course, the higher report frequency) and every 3 min while they are at anchor [10]. Ships over 300 Gross Tonnage (GT) engaged in international voyages, cargo ships more than 500 GT in inland voyages, and all passenger ships regardless of size are required to install an AIS transponder on shipboard by International Maritime Organization [34].

The second dataset is a global ship database which contains the detail information of more than 100,000 vessels all over the world, such as ship MMSI, Call Sign, name, service speed, dimensions, Dead Weight Tons (DWT), GT, and so on. Based on these MMSI, the ship characteristics can be related to the AIS database and then detailed movement and the physical characteristics of each ship.

The third dataset is the World Port Index (WPI), which is published by the National Geospatial-Intelligence Agency (NGA) and publicly available. WPI contains the geolocation, physical characteristics, and facilities and services offered by approximately 3700 major ports worldwide [35]. Many ports, for instance, the Port of Shanghai, consist of several terminals, and the center location of its host city is typically used to represent the port location. In addition, WPI also provides the associated regions and countries of these ports.

The last dataset is the Global Administrative Areas (GADM) database, which is freely available for academic and other noncommercial use, and is mainly maintained by the University of California. The GADM is a spatial database of the geolocation of the administrative boundaries worldwide and contains countries and lowers level subdivisions, such as provinces, cities, and counties. The current versions of GADM includes 386,735 administrative areas. WPI and GADM datasets are mainly leveraged to add semantic meanings to ship stay locations derived from AIS database in order to form a multi-level structure of these locations.

### 3.2. Methodology

**Figure 1:**
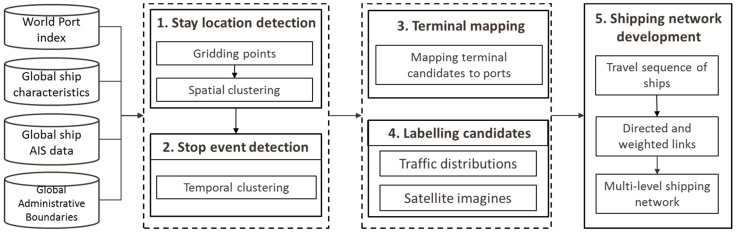
Bottom-up approach for Global Shipping Network (GSN) extraction: Datasets and principles.

The overall process applied to derive a GSN is shown in Figure 1. It mainly consists of five steps. The first three steps generate and construct the network nodes, while the last two steps derive the network links. Specifically, the first step identifies the terminal candidates based on the geographic locations of the ships extracted from AIS datasets. The second step is to detect the stop events of each ship at each terminal candidate according to the temporal characteristics of the AIS data. The next step is to obtain the semantic information of the selected terminals by mapping these terminals to its related ports and countries based on the WPI and GADM reference datasets, which are publicly accessible. The fourth step gives a label to these candidates based on their traffic distribution derived from stop events and satellite imageries and maps offered by service providers. Finally, taking the terminals or ports as nodes and trip statistics between two nodes as links, a directed GSN is finally generated. The rest of this section provides additional details for each of these steps.

#### 3.2.1. Terminal Candidates Identification

Marine terminals are important stay locations that could be extracted from massive historical AIS messages. A first step is to reduce the total number of data points by removing nonsignificant trajectory points. Ships are often static when stopped at terminals, which means that not all AIS points are equally useful. Therefore, we first consider AIS locations whose speed is null and navigation status is equal to “moored” as inputs. Abnormal locations mainly refer to locations with either longitude and latitude irrational values, and implausible locations (i.e., ground locations). They are all detected by the DBSCAN algorithm.

Next, the overall computing time should be minimized. Gridding approaches have been notably used to downsize a given dataset for clustering. This approach is similar to a quadtree algorithm. In order to control the number of points in each grid, the size of the grid can be adapted according to the application context. Specifically, AIS points in the dataset are divided into a set of non-overlapping geographic grids, so that the total number of points in each grid does not go beyond a given threshold (*thre_n*). The ocean surface is initially broken into a set of 1x1 degree grids, then each AIS point is grouped into a grid based on its geolocation. Next, the total number of points of each grid (*grid_n*) is calculated. When a *grid_n* of a given grid is higher than the *thre_n*, this grid is broken into 10 sub-grids. This process does not stop until all grids satisfy the constraint of the *thre_n*. Consequently, all grids will contain a relatively small number of points, with this being an asset for further query manipulations.

Clearly, it intuitively appears that some terminal locations may have an extremely large number of AIS records with similar locations, especially for ships waiting in a given terminal. This shows that the gridding process will never stop when the *thre_n* is set to a value less than that number of points. Therefore, time sampling has been used to reduce the total number of these duplicated locations and to satisfy the gridding requirements. The time sampling method reduces the total number of duplicated points, and this is for two reasons. First, the DBSCAN algorithm usually requires several closed points to form a cluster, namely, to find a stop event. Time sampling is used to ensure a minimum number of points is consider in time to form a cluster. Moreover, as a given ship may visit the same terminal many times in one year, the idea is to also merge AIS points when they denote locations visited several times. For example, time can be modelled as a series of time slots with equal time length, and each point at these locations is grouped into a specific time slot based on its timestamp, while only one point for each slot is retained.

**Figure 2:**
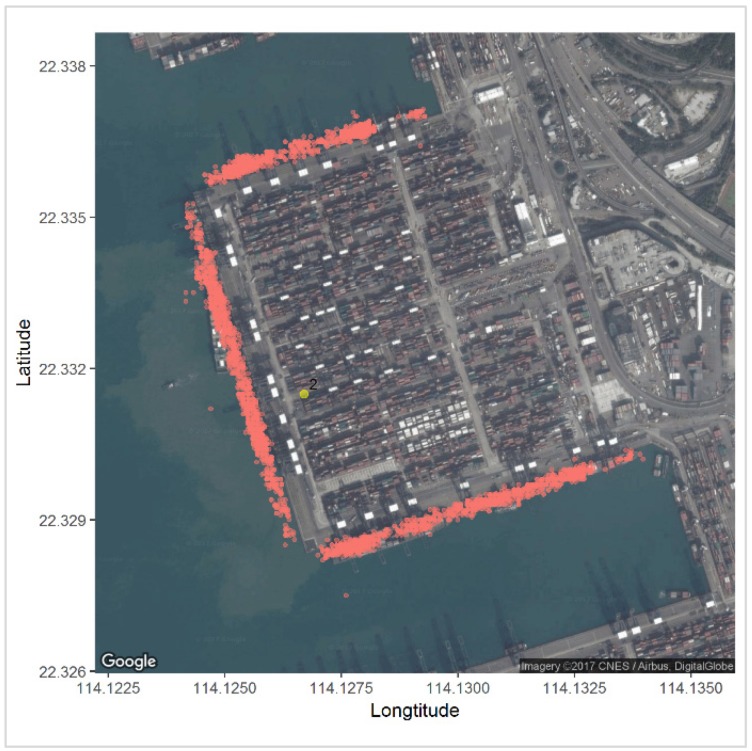
A typical satellite imagery of a container terminal with large number of Automatic Identification Systems (AIS) data points reported from ships then stay at the terminal.

The third step is to identify the terminal candidates at each grid. A typical terminal usually contains several berths and is frequently visited by many ships. Each ship may visit and stay at a terminal for at least several hours to unload or upload cargos, which results in a considerably large amount of AIS messages. Consequently, the density of AIS points at the terminal is likely to be higher, as well as much higher than nearby locations, as illustrated in Figure 2.

DBSCAN is an unsupervised, density-based clustering method particularly developed to detect arbitrary shape from big datasets with noises. It is applied here for terminal identification. The DBSCAN algorithm is fast, robust, and suitable for the mining tasks considered. The main peculiarity of our work lies not only in the multi-level application, but also in the way the DBSCAN parameters are selected. This reflects the fact that the domain knowledge is taken into consideration. These properties are crucial to detect terminal locations, as terminal shapes largely vary from port to port. Moreover, since AIS data are often not 100% reliable and noisy points that can be suppressed, this is a secure property to further develop our project.

It has been observed that AIS data might be not completely reliable when considered at the local and individual levels, and this can happen for many reasons (e.g., communication errors, AIS data not always emitted or received). However, the advantage of our framework is that the large trajectory datasets considered are much more reliable for statistically analyzing the patterns that appear at both the global, regional, and even local scales. Furthermore, the application of the DBSCAN algorithms is noticeably efficient in dealing with large datasets with local noisy data

DBSCAN requires two parameters. The first one denotes the search radius (*eps*) to search for nearby neighbors. The second one refers to the minimum amount of points required (*minPts*) to form a core point. The clustering process of DBSCAN starts with an arbitrary point (*p*) and then searches for points located within the *eps-neighborhood* of *p*, denoted by *N_eps_*(*p*). If the number of points |*N_eps_ (p)*| >= *minPts*, then *p* will be qualified as a core point and a cluster is identified. The algorithm then evaluates the next point and further expands the cluster or develops new clusters until all the points are visited.

Terminal candidates of each grid should be also detected by DBSCAN. Each candidate location is represented by the median location of all the points contained. Although gridding could significantly reduce the total points of each grid, a real terminal may be broken into multiple terminal candidates associated to different grids. These candidates need to be merged back. DBSCAN is employed to merge them back with *eps* = 0.01 (about 1 km) and *minPts* = 1. This means that any two candidates with a distance less than *eps* will be merged.

#### 3.2.2. Stop Events Detection

The purpose of the stop events detection is to identify the stops of each ship at each terminal candidate. On the one hand, statistics on these stops can help to differentiate real terminals from other stay locations. On the other hand, stops could be used to construct the travel sequence of each individual ship. It is common that a ship may spend several hours to travel from one terminal to another while it only takes several minutes to update ship location when stopped at a terminal. This means that the time interval between two terminals is much longer than time gap of any two continuous points within a stop event. Here, and finally, the DBSCAN algorithm is employed to detect stop events according to a temporal perspective.

#### 3.2.3. Terminal Mapping

A terminal candidate identified in the previous step can be simply considered as a set of data points, which are difficult to consider for further analysis due to lack of semantic meanings. Our approach associates these terminals with real ports and countries as identified by WPI and GADM. It would be reasonable to map these terminals with their nearest port based on port locations given by WPI. However, this is not an error-prone approach. For example, locations of the Port of Shanghai and Zhoushan are located at the center of their host cities according to WPI, while the location of the Yangshan, terminal one of the main terminals of the Port of Shanghai, is much closer to the Port of Zhoushan, which leads to several potential mismatches.

**Figure 3:**
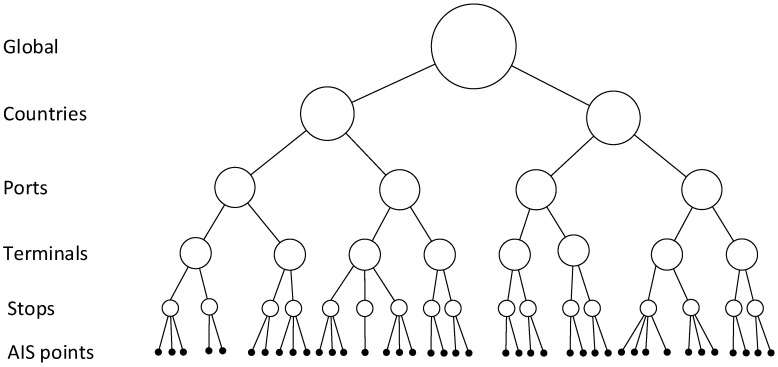
A bottom-up tree-based hierarchical structure of global ship stay locations with incoming AIS data at the bottom level. Global is at the top level, while the stops, terminals, ports, and countries are modelled at the middle levels.

In order to appropriately identify terminal, a three-set process is applied using GADM city boundaries and WPI port terminal locations. First, WPI ports are mapped to corresponding closest cities according to GADM city boundaries. Second, a similar process is applied to terminal candidates. Finally, and as a city can have several ports, each candidate terminal is mapped to the closest port. Overall, this gives a complete bottom-up tree-based hierarchical structure of locations at the global scale as illustrated in Figure 3. From a large set of individual AIS locations and appropriate port and terminal data are associated whenever possible.

#### 3.2.4. Labelling Terminal Candidates

**Figure 4:**
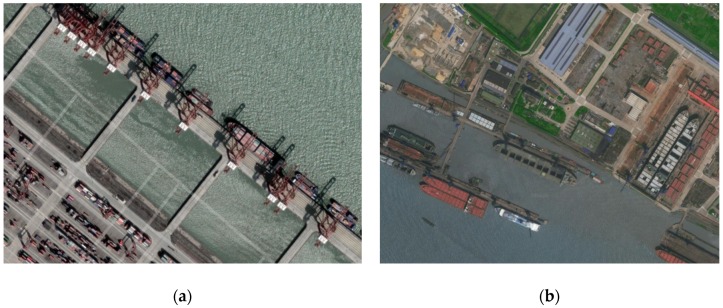
Two different satellite images from terminal candidates. (**a**) Satellite image of a real container terminal. (**b**) Satellite image of a repairing shipyard.

The last step is to label terminal candidates based on satellite images and traffic statistics of each terminal candidates. A terminal candidate may be a real terminal, an anchorage ground, a repairing shipyard, or others. It would be easy to recognize a terminal based on its satellite imagery. For example, a terminal candidate is probably a container terminal if its satellite imagery contains containers or container cranes. Figure 4 shows the difference between two different types of terminal candidate, while the left one is a real container terminal.

At the current stage, a manual approach is applied to classify the terminal candidates. The main process is as follows. First, a satellite image of a terminal candidate is downloaded from map service providers. Second, traffic statistics are derived from stop events at each candidate terminal. Key statistics of a typical terminal candidate include the total number of ships visited, total number of stops, and the duration of these stops. The classification process mainly depends on the analysis of the satellite images, while the statistics provides complementary information. The objective is to detect locations with a high number of ships and stops, as these are likely to be a real terminal. Indeed, future research will be oriented toward the development of a data mining approach to automatically classify these terminal candidates by considering both satellite images and statistical information.

Traffic statistics of a terminal candidate can clearly differentiate it from other types of stop locations since ship behavior and traffic distribution of different stay locations are significantly different. For instance, ship directions change much more frequently at anchorage grounds than at terminals. Ships usually spend more time at repairing shipyards than at terminals. A combination of satellite imageries and traffic information of stay locations provide better accurate labelling.

#### 3.2.5. Network Initialization

Given the stay locations and travel sequences of each ship, a multi-layer shipping network can also be built at different levels of scale. Such networks are generated with stay locations represented as nodes, and ship trips from one node to another as links. The directions of these links start from departure nodes and terminate at destination nodes. The weight of a given link is valued by the sum of the ship capacity of each given trip. This gives a set of directed and weighted shipping networks at multiple scale levels.

## 4. Results and Discussions

The proposed methodology has been applied to different type of ships. Container ships with capacity of more than 1000 TEUs have been considered as an example to develop a Global Container-Shipping Network (GCSN) based on massive ship trajectories in 2015. The global AIS database and ship characteristic database are mainly provided by www.hifleet.com, which is one of the leading maritime data providers in China.

### 4.1. Data Preparation

AIS data of container ships is extracted from the global AIS database by matching the MMSIs of AIS data with the MMSIs of container ships from the global ship database. Overall, this gives 4333 container ships with 349,759,314 AIS points distributed in 23,111 1x1 degree geographic grid cells. Of them, 3593 have a capacity equal to or great than 1000 TEUs, accounting for approximately 84% of the total capacity of the world container ship fleet. With the purpose of mining marine terminals, data points with a speed of 0 and status of “moored” have been selected, which results in 27,106,564 AIS points located in 635 1x1 degree geo-grids.

Although the size of the original dataset has been considerably reduced by the above process, two tasks are taken to facilitate the DBSCAN data analysis. The first one is a time sampling operation. It appears that 147 geo-points have more than 2000 duplicated points (i.e., with same latitude and longitude). Time sampling is therefore used to reduce these duplicated points. Only one point is retained for every 5 min for each of these geo-points for each ship. Finally, 26,881,581 data points were generated after this data filtering process. Therefore, all data points were grouped into appropriate grids. These grids are initialized with different sizes depending on their geo-locations. The value of *thre_n* depends on the size of the host computer’s RAM and speed required to run DBSCAN. Computing the distance matrix for unsupervised clustering like DBSCAN is time-consuming for large datasets and requires very large RAM for storage, as the distance matrix is square the number of points. As performed by sequential computing (a parallel computing solution being left for further work) the grid threshold *thre_n* has been experimentally set to 50,000. Grids will continue to break into 10 small grids until all grids contain no more than *thre_n* points. Finally, 5404 grids of different sizes were generated.

### 4.2. Terminal Detection

The objective of the DBSCAN process is to identify the container terminals for each grid. Given the large number of grids, two R packages have been implemented to faster the DBSCAN clustering process. The first one applies a kd-tree data structure in order to faster the k-nearest neighbor search. The second one enables parallel execution, namely, it can execute those repeated operations on multiple cores from a local computer or on multiple nodes of a computing cluster.

Selecting suitable *eps* and *minPts* is an important step for the terminal detection. The *eps* is set to 0.005, which is about 500 m, considering that the length of a terminal usually ranges from several hundred to several thousand meters. Although some terminals such as the Yangshan terminal of the Shanghai Port is much longer than 500 m, it still can be identified by DBSCAN as the distance between two sequential AIS points within a terminal is much lower than 500 m.

The parameter of *minPts* is related to the total number of AIS points a ship produces. According to [10], ships normally send AIS message every 3 min when the speed is less than 3 knots. Logically, a static ship at a terminal will produce approximately 20 AIS points every hour. Considering that any ship stays at a given terminal more than once, and that a terminal is very likely to be visited by several ships for a considered and sufficient large period of time, this should give a minimum of 20 AIS locations for that terminal.

However, AIS data are not always perfectly collected due to inappropriate manual AIS setup, poor AIS coverages in small terminals, or message losses during a transmission process. With a value of 10 given to *minPts*, this generates 3986 stay locations at 3706 grids. It is noteworthy that a container terminal may be separated by multiple stay locations within an individual grid or different grids. Therefore, these stay locations are further merged by a DBSCAN algorithm with *eps* = 0.01 (i.e., about 1 km) and *minPts* = 1, which means that any two stay locations with a distance less than *eps* merges together to form a new stay location. This process goes over all stay locations until none of them could be merged and then terminates. Finally, 998 terminal candidates were identified.

DBSCAN are leveraged to identify the stop events of each ship in every terminal candidate, and by taking into account a temporal perspective. The *eps* is set to one hour, since the time gap between two sequential visits of a terminal usually take at least several hours or days. The *minPts* is set to 2 points in order to capture terminal stop events with a few points in one hour caused by various AIS coverage and transmission issues. In total, we detected 252,556 stop events for 2015.

These terminal candidates are then labelled according to the previously downloaded satellite images. Additionally, the distribution of the stay duration for each candidate terminal is derived from their associated stops, and this is done for all available candidate terminals. This produces and identifies 754 container terminals. It is to be noted that some small container terminal may be missed out due to few container visits and poor AIS coverage. However, these few small container terminals do not have a significant impact on the structure and characteristics of the global container shipping network.

With the purpose to add the semantic meaning of these stay locations and increase the convenience of further analysis, WPI and GADM are employed to link the identified container terminals to their associated ports and countries based on their geographic locations. Finally, 513 associated ports from 150 countries are identified from WPI data.

### 4.3. Shipping Network

**Figure 5:**
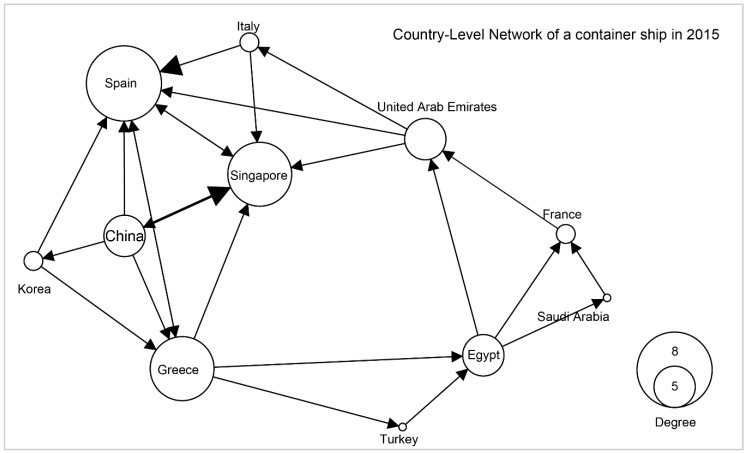
Country-level network of a container ship with 11 nodes in 2015.

**Figure 6:**
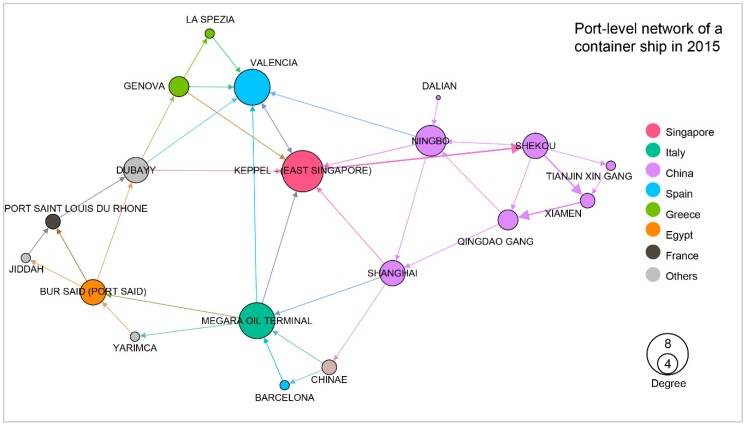
Port-level network of a container ship with 19 nodes located in 11 countries in 2015.

**Figure 7:**
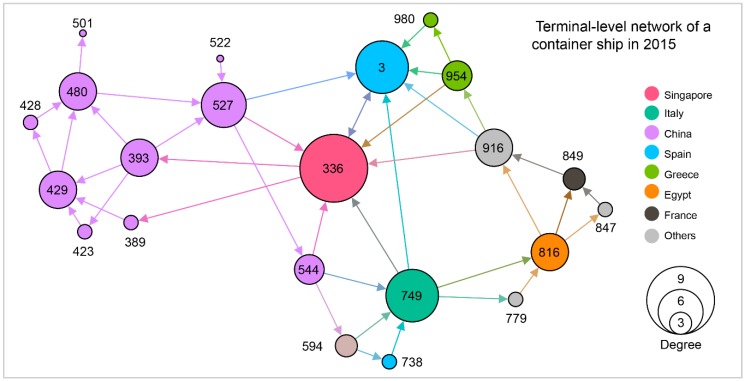
Terminal-level network of a container ship with 22 nodes located in 11 countries in 2015.

Travel sequences of each ship could be viewed as a small network where nodes denote either terminals, ports, or countries the ship visited, while links denote direct travels between these ports. The weight of each link is given by the sum of the TEUs of its associated travels. Let us consider a typical container ship with a capacity of 13,798 TEU as an example to build different network levels. Figure 5, Figure 6 and Figure 7, respectively, denote the country, port and terminal-levels. All three networks are weighted represented as directed network. As shown in this example, this ship visited 11 countries, 19 ports, and 22 terminals, and overall mainly shows routes between Asia and Europe.

**Figure 8:**
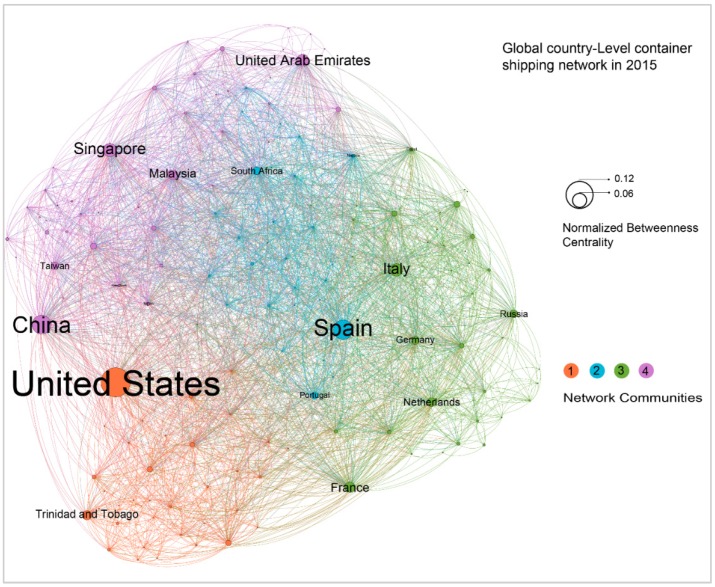
Global country-level container shipping network in 2015. A directed network with 2842 links and 150 countries. The size of a node refers the normalized betweenness centrality of a given node. The color refers to the four community clusters of the network. The color of a link is equal to the color of its departure node.

**Figure 9:**
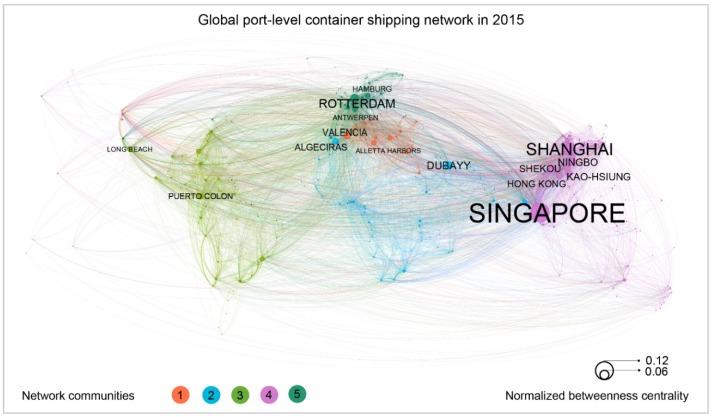
Port level Global Container Shipping Network (GCSN) extracted from ship AIS data in 2015. A directed and weighted network with 9227 links and 513 port nodes located in 150 countries. The size of a node refers the normalized betweenness centrality of a given node. The color refers to the five community clusters of the network. The color of a link is equal to the color of its departure node. The width of a link is equal to a rescaled link weight.

**Figure 10:**
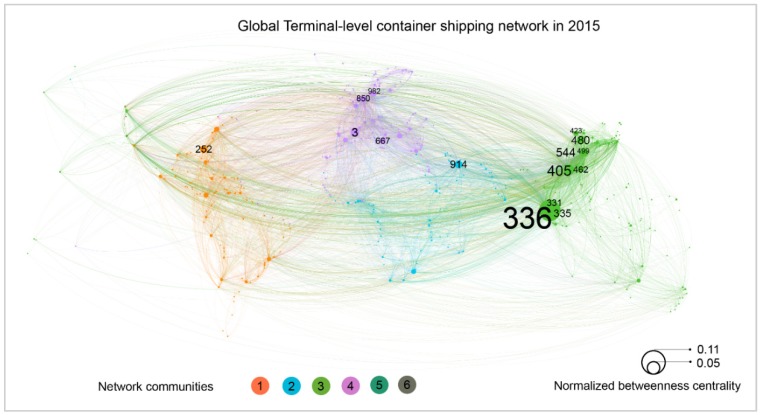
Terminal level GCSN extracted from ship AIS data in 2015. A directed and weighted network with 14,060 links and 754 container terminal nodes located in 513 ports. The size of a node refers the normalized betweenness centrality of a given node. The color refers to the six community clusters of the network. The color of a link is equal to the color of its departure node. The width of a link is equal to a rescaled link weight. The label of each node is the global terminal ID produced by this study.

The GCSNs could be viewed as a potential merge of these relatively small networks produced by individual ships at some given levels. Taking the port-level network as an example, we developed a GCSN at the port level by integrating the port level of networks from all ships and viewing ports as nodes and direct journeys between two ports as links. A weight is also assigned to each link, which is equal to the sum of TEUs of each ship journey on the link in 2015. Accordingly, we developed three global container shipping networks as shown in Figure 8, Figure 9 and Figure 10 at the country, port, and terminal levels, respectively. Figure 8 shows that mainland China to Hong Kong, Singapore to mainland China, Hong Kong to mainland China are the top three links. Moreover, the United States, China, and Spain are the most important hubs at the country level. This reflects large-scale trades among the United States, China and Europe. However, at the port level, it appears that the ports of Singapore, Shanghai, and Rotterdam are the top world hubs.

All of these networks are developed by Gehpi, an open network analysis and visualization software. The main process of developing the figure includes three steps. First, port-level nodes with port names and geo-coordinates, and links with departure and destination port nodes and weight in TEUs are loaded into the software. Second, embed network analysis functions of Gephi are leveraged to calculate the total degrees, betweenness centrality, and the network community of each node. The last step is visualization. For network nodes, a geo-layout is used to locate nodes to their geo-coordinates. Node sizes are equal to their normalized betweenness centrality, node colors are equal to their associated network communities, and the labels of nodes are their port names derived from WPI. The sizes of these labels are proportionate to the size of their nodes. For links, the direction of each link is from original port to destination port derived from the its associated ship trips. The width of a links is set to its normalized weight, which is calculated by summing of ship capacity (TEUs) of each trip in the link. The color of each link is same to the color of its original node.

The characteristics of each network level have been derived and compared (see Table 1). The main features include the number of nodes and links, network diameter, modularity, and average path length. These figures are derived from the country to the terminal level networks and overall show some decreasing patterns. In contrast, the value of average clustering coefficient increases while the average degree is relatively stable. These differences reflect the properties of the emerging networks at high scales.

For port-level network, the average shortest path length is 2.77. This is in agreement with the work of Kaluza et al. [6], which finds the average shortest path length is 2.76. The node locations are the geo-location and labels of the ports adapted from WPI, while the size of the nodes represent the normalized betweenness centrality of these nodes, and which quantifies how often a node appears on the shortest paths between the nodes in the network. In other words, this reflects the hub function of a node. As shown in the figure, the ports of Singapore, Shanghai, and Rotterdam are the top three container shipping hub ports worldwide in 2015. In addition, this study also investigates the communities of the GCSN while taking the link weight into account. Five network community clusters are identified. As indicated by the figure, cluster 3, 2, and 1 are the top three communities, accounting for 29.4%, 24.4%, and 17.5% of the total number of nodes, respectively. We also found that the top three links are Ningbo to Shanghai, Singapore to Shekou, and Shekou to Hong Kong.

Overall, it appears that a terminal-level network has much more nodes and links than the one of a port-level network. Furthermore, a terminal-level network has an average shortest path length of 2.90 and network diameter of 8, which is higher than the one of a port-level network. This may be caused by the higher number of nodes and links for terminal-level networks, and by local cross-terminal transportations within an individual port. Furthermore, it also appears that betweenness centrality values are stable for Singapore, which is still the most important hub node either in terminal level or port level. However, the ports of Shanghai and Rotterdam, which rank the second and third place at port-level network, are dropped out from the top three positions. This is mainly due to the fact that Shanghai and Rotterdam consist of more geo-separated terminals and by the fact that the hub function is distributed between different terminals. Consequently, the ports of Shanghai and Rotterdam are respectively ranked the fourth and twelfth for the terminal-level hub function. Conversely, a terminal of Hong Kong becomes the second hub terminal worldwide. The fact that the method is very successful in identifying stay terminals, main routes and associate speed profiles between them provides additional processing capabilities for evaluating fuel consumption. In a previous work [36], a data mining approach was applied to identify speed profiles for evaluating fuel consumptions.

## 5. Conclusions

This study explores the possibility of constructing a GSN based on large ship AIS sensor data. A bottom-up data-driven approach is developed based on a tree-based structure of multi-level global stay locations and shipping networks. A combination of a clustering and semantic approach is applied and further extends the previous port-level shipping networks to the terminal-level and country-level networks. This allows us to identify marine terminals and their associated ship stops extracted from individual AIS data points. The proposed approach is successfully applied to the development of a global container shipping network (GCSN) with multiple spatial levels. The main features, such as average degree and betweenness centrality of each node, average shortest path length between any two nodes, and community clusters of the GCSNs, are derived and carefully compared.

The aim of our study is to provide an AIS data-driven approach to construct a global shipping network with the container shipping network as an example to illustrate the method. However, this method is also applicable to tramp shipping. Our approach is not only suitable for an analysis of container traffic flows or world trade analysis, but also for lower shipping-behavior-level analysis, since AIS trajectories between two nodes can be analyzed at a finer level of granularity.

This work can be, however, extended in several directions. A first one is to improve the identification of terminal locations. Machine learning methods might be explored and applied to automatically identify terminals based on several complementary factors, such as locations, satellite images, and traffic statistics at each stay locations to accelerate the terminal detecting process. Another one is to optimize the parameters of DBSCAN. Although our study applies similar parameters for each grid, an application of customized parameters for each grid might better reflect the different densities and then improve clustering results. It is also notable that container ships practically always call at more than two ports with several transshipments. Current extracted data may be difficult to identify this kind of connections, so additional work still need to be explored using data mining methods to detect repeated paths or integrating shipping schedule data. This is also a direction to explore in further work using a combination of statistical and network analysis. Finally, the proposed method can be also applied to different types of ships in order to generate a global shipping network.

## Figures and Tables

**Table 1:** Characteristics of three different levels of global container networks.

Networks	Number of Nodes	Number of Links	Average Degree	Network Diameter	Modularity	Average Clustering Coefficient	Average Path Length
Terminal level	754	14,060	18.647	8	0.37	0.365	2.90
Port level	513	9227	18.986	7	0.35	0.409	2.77
Country level	150	2842	18.947	5	0.24	0.523	2.24
